# Extracellular vesicles-based pre-targeting strategy enables multi-modal imaging of orthotopic colon cancer and image-guided surgery

**DOI:** 10.1186/s12951-021-00888-3

**Published:** 2021-05-22

**Authors:** Boping Jing, Ruijie Qian, Dawei Jiang, Yongkang Gai, Zhen Liu, Feng Guo, Sen Ren, Yu Gao, Xiaoli Lan, Rui An

**Affiliations:** 1 Department of Nuclear Medicine, Union Hospital, Tongji Medical College, Huazhong University of Science and Technology, No. 1277 Jiefang Ave, Wuhan, 430022 Hubei Province China; 2 Department of Pancreatic Surgery, Union Hospital, Tongji Medical College, Huazhong University of Science and Technology, Wuhan, 430022 China; 3 Department of Hand Surgery, Union Hospital, Tongji Medical College, Huazhong University of Science and Technology, Wuhan, 430022 China

**Keywords:** Extracellular vesicles, PET/CT, NIRF, Multimodal imaging, Image-guided surgery

## Abstract

**Backgroud:**

Colon cancer contributes to high mortality rates as the result of incomplete resection in tumor surgery. Multimodal imaging can provide preoperative evaluation and intraoperative image-guiding. As biocompatible nanocarriers, extracellular vesicles hold great promise for multimodal imaging. In this study, we aim to synthesized an extracellular vesicles-based nanoprobe to visualize colon cancer with positron-emission tomography/computed tomography (PET/CT) and near-infrared fluorescence (NIRF) imaging, and investigated its utility in image-guided surgery of colon cancer in animal models.

**Results:**

Extracellular vesicles were successfully isolated from adipose-derived stem cells (ADSCs), and their membrane vesicles were observed under TEM. DLS detected that the hydrodynamic diameters of the extracellular vesicles were approximately 140 nm and the zeta potential was − 7.93 ± 0.24 mV. Confocal microscopy showed that extracellular vesicles had a strong binding ability to tumor cells. A click chemistry-based pre-targeting strategy was used to achieve PET imaging in vivo*.* PET images and the biodistribution results showed that the best pre-targeting time was 20 h, and the best imaging time was 2 h after the injection of ^68^ Ga-L-NETA-DBCO. The NIRF images showed that the tumor had clear images at all time points after administration of nanoparticles and the Tumor/Muscle ratio peaked at 20 h after injection. Our data also showed that both PET/CT and NIRF imaging clearly visualized the orthotopic colon cancer models, providing preoperative evaluation. Under real-time NIRF imaging, the tumor location and tumor boundary could be clearly observed.

**Conclusions:**

In brief, this novel nanoprobe may be useful for multi-modal imaging of colon cancer and NIRF image-guided surgery. More importantly, this study provides a new possibility for clinical application of extracellular vesicles as nanocarriers.

**Graphic Abstract:**

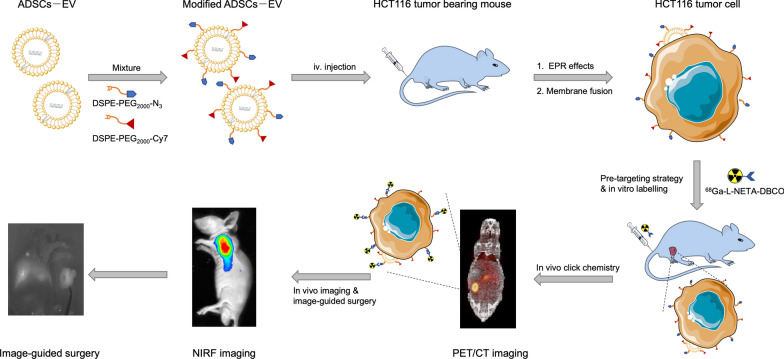

**Supplementary Information:**

The online version contains supplementary material available at 10.1186/s12951-021-00888-3.

## Introduction

At present, surgery still plays a crucial role in colon cancer treatment, with more than 50% of cancer patients undergoing surgery. Complete resection is a major challenge in tumor surgery [1]. In response, multi-modal imaging has been performed to visualize cancer before and during surgical procedures to provide pre- or intra-operative disease-specific images in real time [2]. Multi-modal imaging is a combination of two or more imaging modalities that offer biologically complementary information and may provide a better imaging solution than individual technologies [3]. Positron emission tomography/computed tomography (PET/CT) imaging can enable visualization of tumors and their regression or progression, offering high sensitivity, excellent penetration, and high spatial resolution [4]. Near-infrared fluorescence (NIRF, 650–1000 nm) imaging can provide anatomical information and real-time delineation of tumor because of the advantages of high temporal resolution, spatial resolution, super sensitivity and lower background [5–7]. Therefore, multi-modal PET/CT and NIRF imaging of colon cancer can be applied to obtain preoperative and intraoperative imaging of tumor.

Nanoparticle-based tracers have unique characteristics to serve as carriers for both radionuclide and NIRF dye labels during image-guided surgery. Recently, extracellular vesicles have attracted increasing attention, as they play important roles in physiology, pathology, and oncology. Extracellular vesicles are nanosized phospholipid bilayer vesicles secreted by various cells [8]. As biological nanoparticles, extracellular vesicles have been used to deliver drugs to specific cell types or tissues in vivo, especially tumor tissues [9–11]. For example, drugs like paclitaxel, imatinib, doxorubicin, curcumin, anthocyanidin, acridine orange, as well as nucleic acids, have been successfully loaded into extracellular vesicles and delivered to target cells [12]. Previous studies have confirmed that extracellular vesicles are ideal nanoparticles because of the following advantages: they are secreted by nearly all cell types and can be found in multiple types of extracellular fluids, such as the blood, urine, amniotic fluid, saliva, cerebrospinal fluid, and breast milk [13]. Second, the intrinsic small sizes of some extracellular vesicles facilitate their extravasation through tumor vessels and their subsequent diffusion into tumor tissues [14]. Third, the membrane structure of extracellular vesicles is similar to that of cells. They can be easily modified with functional groups and directly fuse with the membrane of target cells, thus improve the feasibility of modifying the target cell membrane [15]. Some human clinical trials performed using extracellular vesicles from dendritic cells for cancer therapy reported positive results regarding their feasibility and safety [16]. As reported, extracellular vesicles from various sources have different properties and can be applied to distinct functions [17]. Adipose-derived stem cells emerged as a stable source of extracellular vesicles due to the advantages of facile availability. Extracellular vesicles extracted from adipose-derived stem cells (ADSCs-EV) can be employed to engineer a multi-modal imaging probe because of their preferential tumor targeting ability [17].

To date, great efforts have been dedicated to investigating their applications as natural drug-delivery systems, but the tracing of extracellular vesicles has not been perfected, which stands to accelerate the clinical applications of extracellular vesicles-based drug delivery systems and the study of extracellular vesicles-based theranostic probes. Previous studies have traced extracellular vesicles-based drug delivery systems by optical imaging [18], but it was neither quantitative nor accurate because of the limited tissue penetration. Magnetic resonance (MR), PET, and single photon-emission computed tomography (SPECT) have been applied for tracking extracellular vesicles and analyzing their biodistribution [19–22]. Therefore, the possibility of extracellular vesicles used as nanocarriers for multi-modal imaging of colon cancer has been demonstrated in theory. However, most previous studies exhibited high tracer uptake in the liver, spleen, and kidneys, and low uptakes in tumor sites, making it difficult to compare the biodistribution of extracellular vesicles from different sources in vivo from images.

**Scheme 1:**
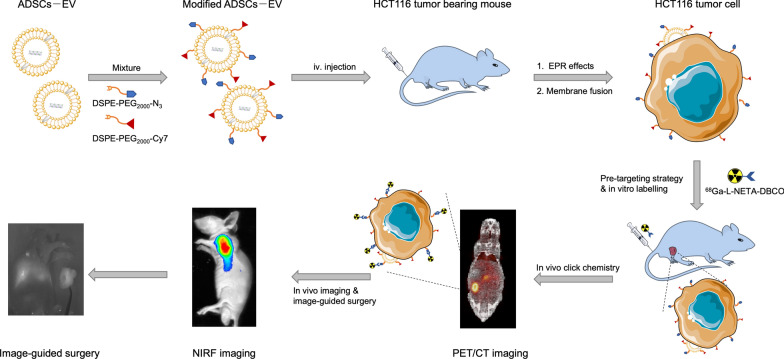
The schematics of multimodal PET and NIRF imaging and real-time NIRF intra-operation based on extracellular vesicles from ADSCs

In this study, extracellular vesicles were isolated from ADSCs and modified with 1,2-distearoyl-sn-glycero-3-phosphoethanolamine-N-[azido(polyethyleneglycol)-2000] (DSPE-PEG_2000_-N_3_) and DSPE-PEG_2000_-Cy7, then the modified ADSCs-EV (N_3_-EV-Cy7) was obtained. A pre-targeting strategy are applied to label N_3_-EV-Cy7 for imaging with the goal of increasing the target/non-target ratio and improving the image quality. Additionally, the strategy can shorten imaging time and reduce potential radiation damage. Then, in vivo PET/CT and NIRF imaging of tumor-bearing nude mice was performed to verify the potential application of extracellular vesicles. Subsequently, tumor resections were performed to under real-time NIRF imaging (Scheme 1). We believe our results confirm the feasibility of extracellular vesicles-based nanoprobes for multimodality imaging and image-guided surgery of colon cancer in animal models.

## Results

### Characteristics of ADSCs-EV

**Fig. 1:**
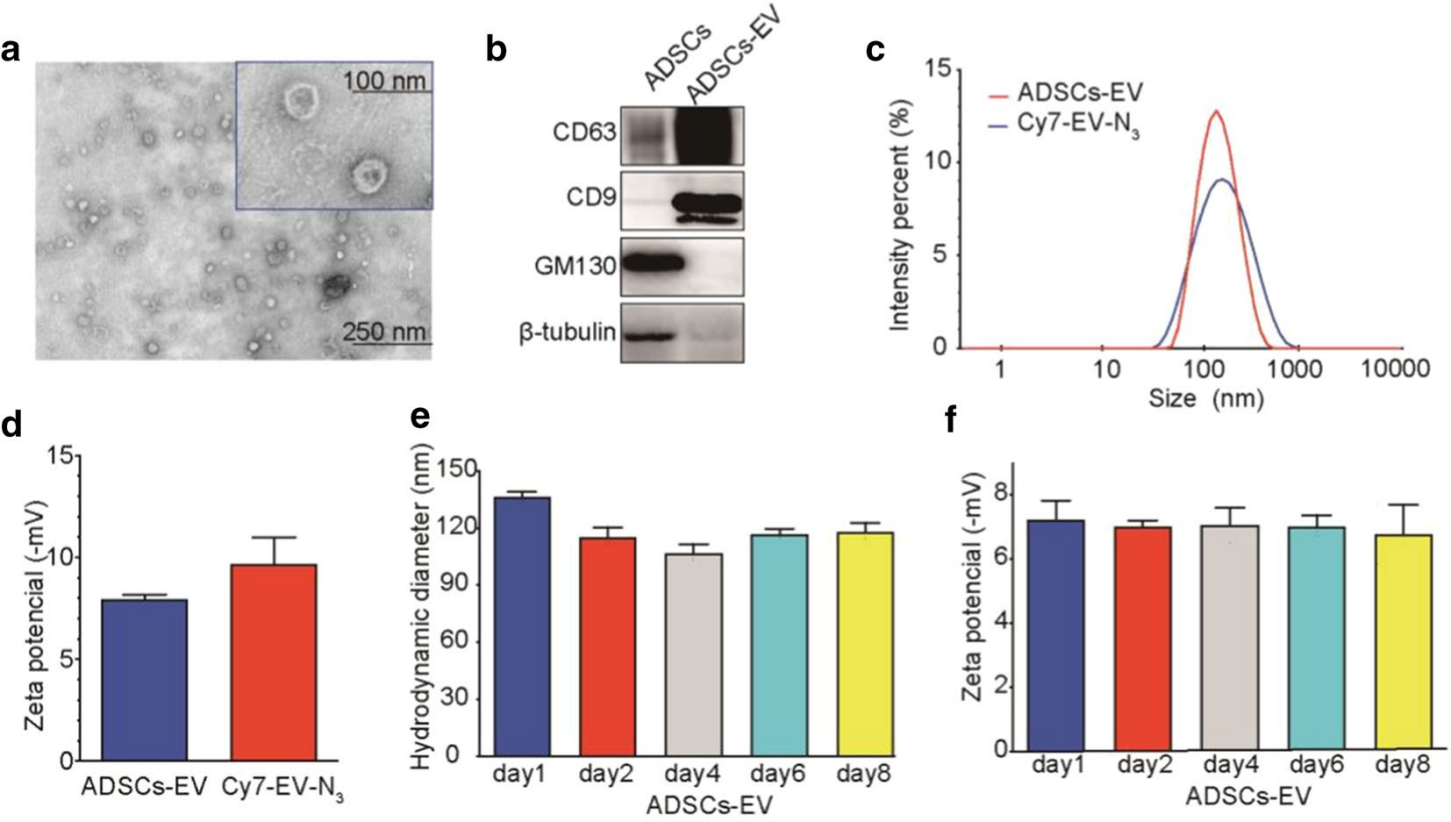
The identification and characteristics of ADSCs-EV. **a** Membrane vesicles were observed under Transmission Electron Microscope (TEM). **b** Western blot confirmed the expression of ADSCs-EV markers (CD63, CD9). **c** The average hydrodynamic diameters of ADSCs-EV and Cy7-EV-N3. **b** The zeta potential of ADSCs-EV and Cy7-EV-N_3_. **e** The changes of hydrodynamic diameters of ADSCs-EV at 4℃ over 8 days. **f** The changes of zeta potential of ADSCs-EV at 4℃ over 8 days

ADSCs-EV appeared membrane vesicles under TEM (Fig. 1a). Western blotting indicated that the ADSCs‐EV positively expressed surface markers such as CD9 and CD63. While non‐expression markers of ADSCs-EV, such as GM130 and β‐tubulin (Fig. 1b), were negatively expressed. The mean hydrodynamic diameters of ADSCs-EV and Cy7-EV-N_3._ were approximately 140 nm (Fig. 1c). The zeta potentials of the ADSCs-EV and Cy7-EV-N3 were − 7.93 ± 0.24 mV and − 9.68 ± 1.3 mV, respectively (Fig. 1d). There was no significant difference in hydrodynamic diameters and zeta potential between ADSCs-EV and Cy7-EV-N_3._ The stability of ADSCs-EV at 4 °C was great over 8 days (Fig. 1e, f). HCT116 cancer cells or adipose stem cell were co-incubated with ADSCs-EV at various concentrations (up to 100 μg/mL) and different time periods (up to 72 h). The results showed that the survival rate of cells in each group was greater than 90% (Additional file 2: Table S1-S2). ADSCs-EV had no obvious toxicity to HCT116 colon cancer cell and adipose stem cell.

### In vitro tumor cell binding

**Fig. 2:**
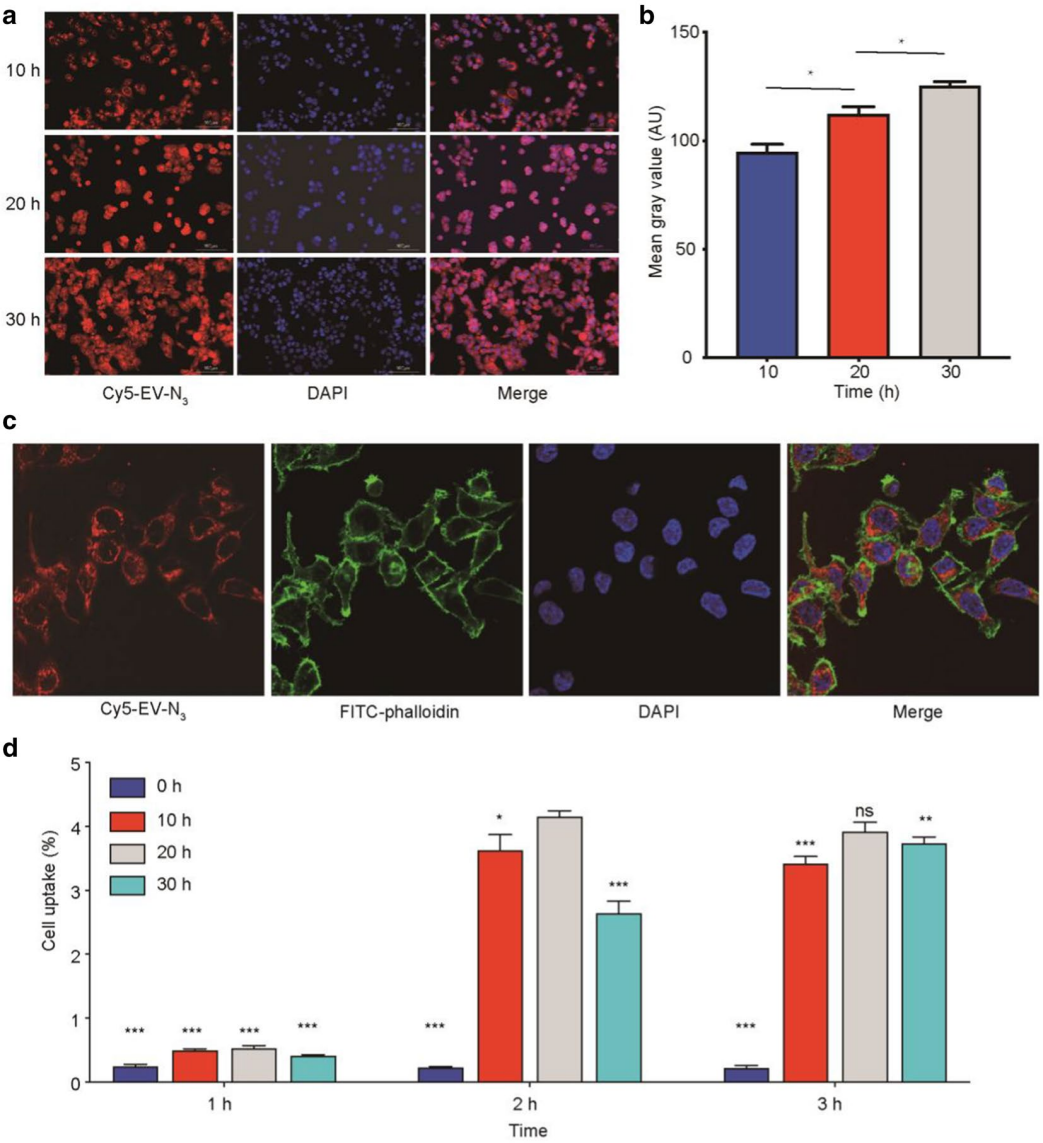
Tumor-binding ability of Cy5-EV-N_3_ and cell uptakes of Cy7-EV-N_3_ with different incubation time. **a** Fluorescence images of HCT116 cells after incubating with Cy5-EV-N_3_ for different time points (200 ×). **b** Corresponding quantification of the fluorescent intensity. **c** Tumor-binding ability was detected by confocal imaging (600 ×). **d** Uptakes of ^68^ Ga-L-NETA-DBCO in HCT116 tumor cells (incubated with Cy7-EV-N_3_ for different time periods) at the indicated time points. Bars represent means ± SD (n = 3). *P < 0.05; **P < 0.01; ***P < 0.001

As exhibited in fluorescence images, the uptake of Cy5 and N_3_ labeled ADSCs-EV (Cy5-EV-N_3_) in HCT116 cell was increased over time (Fig. 2a). The quantification of the fluorescent intensity was consistent with images (Fig. 2b). Confocal microscopy of HCT116 cells incubated with Cy5-EV-N_3_ revealed strong fluorescence signals in the cell membrane and cytoplasm (Fig. 2c). L-NETA-DBCO was used as a bifunctional chelator in this study. The labeling procedure for ^68^ Ga-L-NETA-DBCO resulted in high radiochemical purities (> 95%; Additional file 2: Figure S1A). As shown in Additional file 2: Figure S1B, the proportion of intact tracer exceeded 90% after incubation in serum at 37 °C for 2 h, indicative of excellent serum stability of our tracer. HCT116 cancer cells or adipose stem cell were co-incubated with ^68^ Ga-L-NETA-DBCO (37KBq/well) at different time periods (up to 72 h). The results showed that the survival rate of cells in each group was greater than 90% (Additional file 2: Table S3). The cell uptakes were displayed in Fig. 2d. The uptake rate of ^68^ Ga-L-NETA-DBCO in HCT116 cells peaked at 2 h after Cy7-EV-N_3_ incubated with HCT116 cells for 20 h.

### In vivo animal PET imaging

**Fig. 3:**
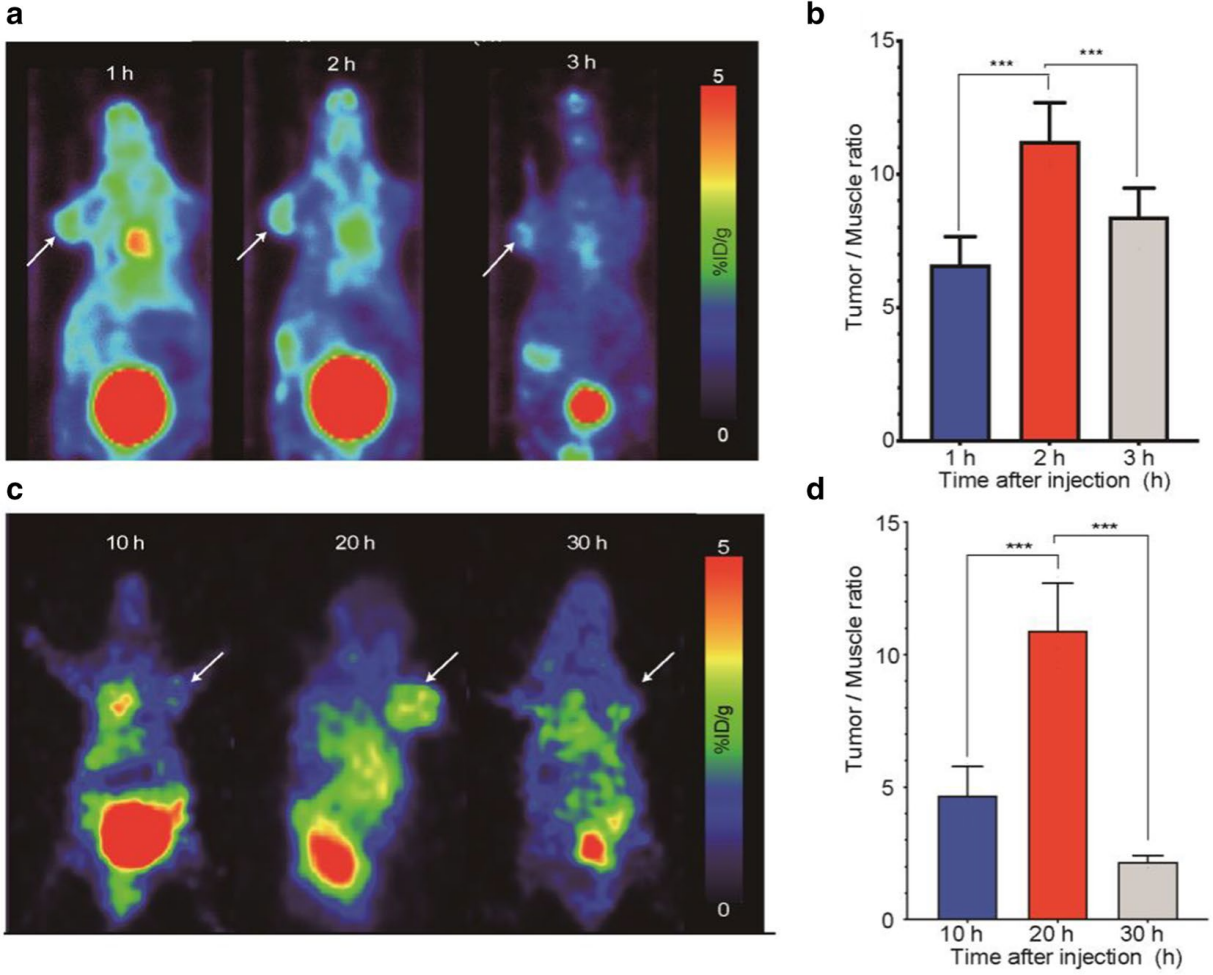
In vivo PET imaging of HCT116 tumor-bearing mice at different pre-targeting time points. **a** Representative static PET images after 1 h, 2 h and 3 h p.i. of ^68^ Ga-L-NETA-DBCO. **b** T/M ratios of different time (1 h, 2 h and 3 h after the injection of ^68^ Ga-L-NETA-DBCO). **c** Representative static PET images after 2 h p.i. of ^68^ Ga-L-NETA-DBCO with different pre-targeting time. **d** Tumor/muscle ratios of different pre-targeting time

Having confirmed the labeling of ^68^ Ga-L-NETA-DBCO, we performed PET imaging based on different imaging time periods (1, 2, and 3 h) and different pre-targeting time periods (10, 20, 30 h). Comparing different imaging time points, maximum tumor uptake was obtained at 2 h after the injection of ^68^ Ga-L-NETA-DBCO (Fig. 3a). The T/M ratios displayed in Fig. 3b were consistent with the PET imaging. Then PET imaging with different pre-targeting time points (10, 20, 30 h) was performed at the best imaging time (2 h). Comparing different pre-targeting time points, PET imaging results indicated that maximum tumor uptake was displayed at 20 h after the injection of Cy7-EV-N_3_ (Fig. 3c). The tumor-to-muscle ratios (T/M ratios) were maximal at 20 h after the injection (11.41 ± 1.78; Fig. 2d). The chelator group (only injected with ^68^ Ga-L-NETA-DBCO) did not show any obvious signal at the tumor site (Additional file 2: Figure S2).

### In vivo biodistribution

**Fig. 4:**
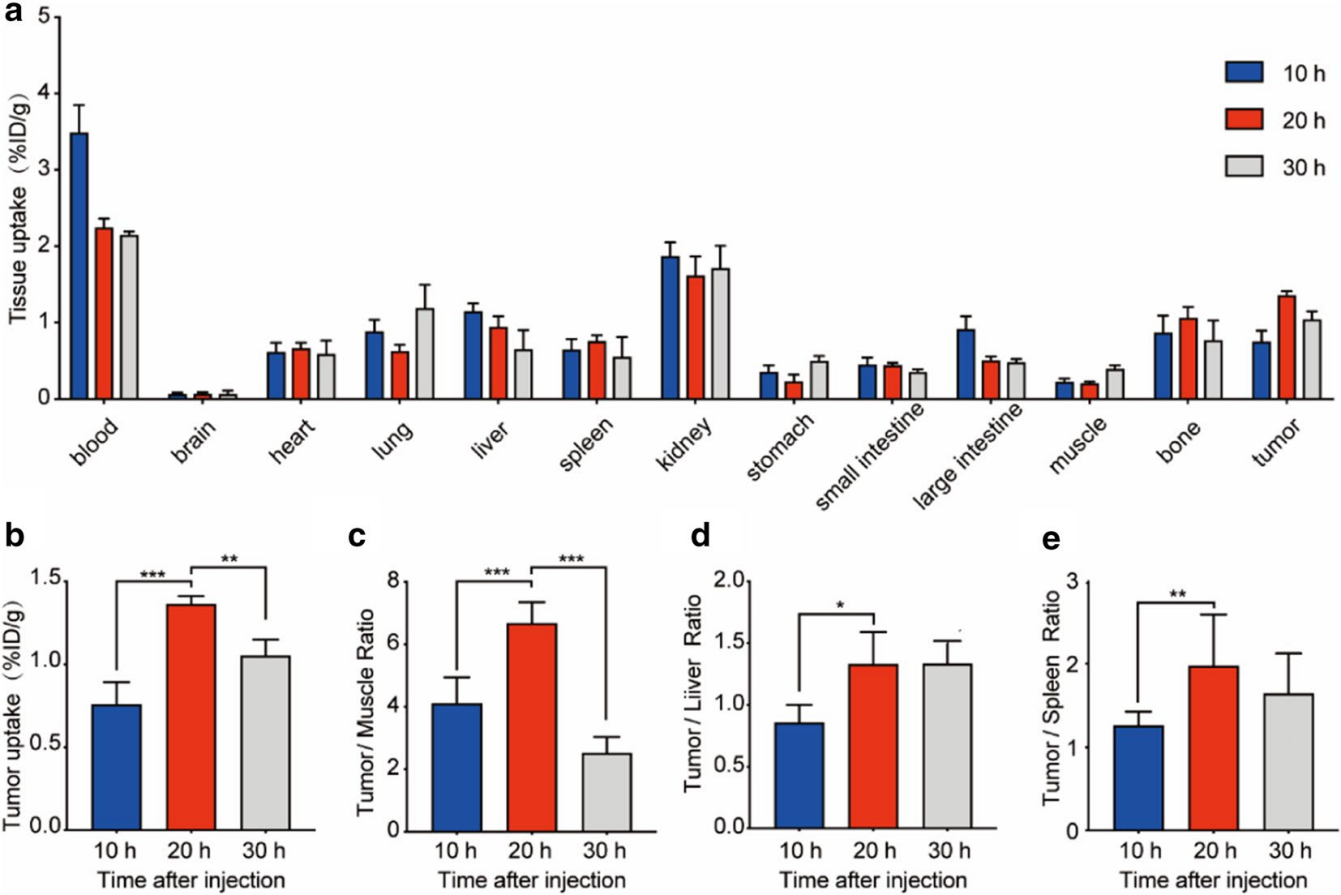
Biodistribution analysis at different pre-targeting time points (10 h, 20 h, and 30 h). **a** Tissues uptakes of HCT116 tumor-bearing mice at 2 h p.i. of 6^8^ Ga-L-NETA-DBCO with different pretargeting time. **b** Tumor uptakes of different pretargeting time points. **c**–**e** Tumor/Muscle ratios, Tumor/Liver ratios and Tumor/Spleen ratios of different pretargeting time points. All bars represent as means ± SD (n = 4). *P < 0.05; **P < 0.01; ***P < 0.001

Biodistribution was conducted to further quantitative analysis of the distribution of this nanoprobe in vivo. The data (Table 1) was consistent with the imaging. Tissue uptakes at different pre-targeting time are shown in Fig. 4a. Tumor uptake (1.37 ± 0.05%ID/g; Fig. 4b) and T/M ratio (6.68 ± 0.68; Fig. 4c) peaked at 20 h after injection. Liver and spleen showed minimum tracer uptake with values lower than 1%ID/g at all time points. The tumor/liver ratio (1.329 ± 0.26; Fig. 4d) and tumor/spleen ratio (1.71 ± 0.16; Fig. 4e) were peaked at 20 h after injection.

**Table 1:** The biodistribution at different pre-targeting time points (10, 20 and 30 h). Data are expressed as mean ± standard deviation (%ID/g, n = 4)

Tissue	^68^ Ga-EV-Cy7		
	10 h	20 h	30 h
Blood	3.36 ± 0.40	2.25 ± 0.11	1.89 ± 0.53
Brain	0.07 ± 0.01	0.08 ± 0.01	0.07 ± 0.04
Heart	0.62 ± 0.11	0.67 ± 0.07	0.53 ± 0.21
Lung	0.89 ± 0.15	0.63 ± 0.08	0.75 ± 0.36
Liver	1.16 ± 0.10	0.95 ± 0.13	0.66 ± 0.24
Spleen	0.66 ± 0.13	0.77 ± 0.07	0.56 ± 0.25
Kidney	1.60 ± 0.42	1.63 ± 0.24	1.37 ± 0.54
Stomach	0.36 ± 0.08	0.24 ± 0.09	0.44 ± 0.15
Small intestine	0.46 ± 0.09	0.45 ± 0.02	0.36 ± 0.03
Large intestine	0.92 ± 0.17	0.51 ± 0.04	0.48 ± 0.04
Bone	0.88 ± 0.22	1.07 ± 0.14	0.77 ± 0.25
Muscle	0.23 ± 0.04	0.20 ± 0.03	0.40 ± 0.04
Tumor	0.87 ± 0.24	1.37 ± 0.13	1.05 ± 0.10

### In vivo NIRF imaging

**Fig. 5:**
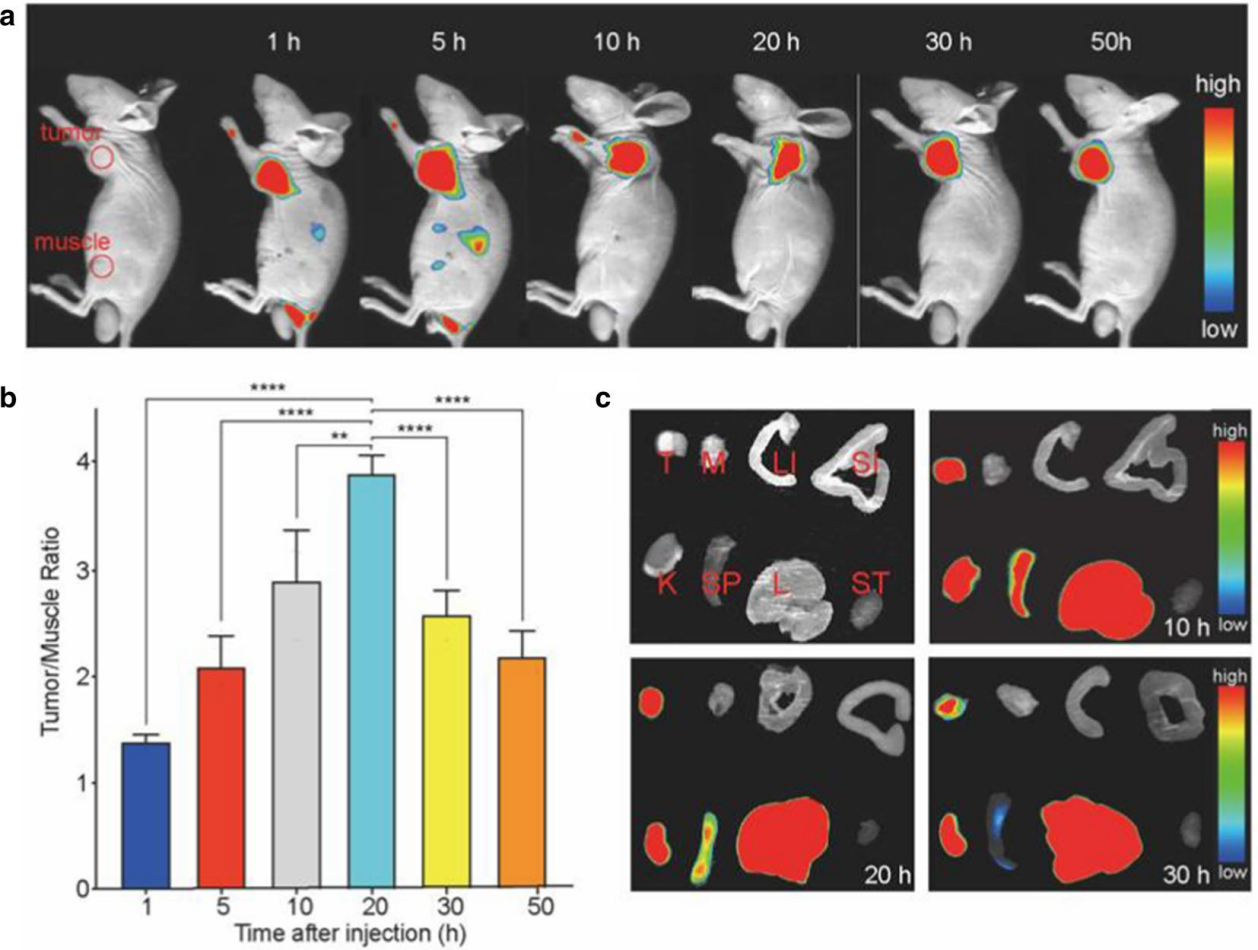
NIRF imaging of HCT116 tumor-bearing nude mice and the tissues. **a** NIRF images of tumor-bearing mice at different time points (1, 5, 10, 20, 30 and 50 h) after the injection of Cy7-EV-N_3_. **b** T/M ratios at different time points after Cy7-EV-N_3_ injection (n = 3, **P < 0.01; ***P < 0.001). **c** NIRF images of ex vivo tissues at different time points after injection (10, 20 and 30 h). *T* Tumor, *M* Muscle, *LI* Large Intestinal, *SI* Small Intestinal, *K* Kidney, *SP* Spleen, *L* Liver, *ST* Stomach

In vivo and ex vivo NIRF images (Fig. 5a and c) indicated that high tumor uptakes were displayed at all time points. The T/M ratio peaked at 20 h after injection (4.63 ± 0.90; Fig. 5b).

### Multi-modal animal PET/CT and NIRF imaging of orthotopic colon cancer

**Fig. 6:**
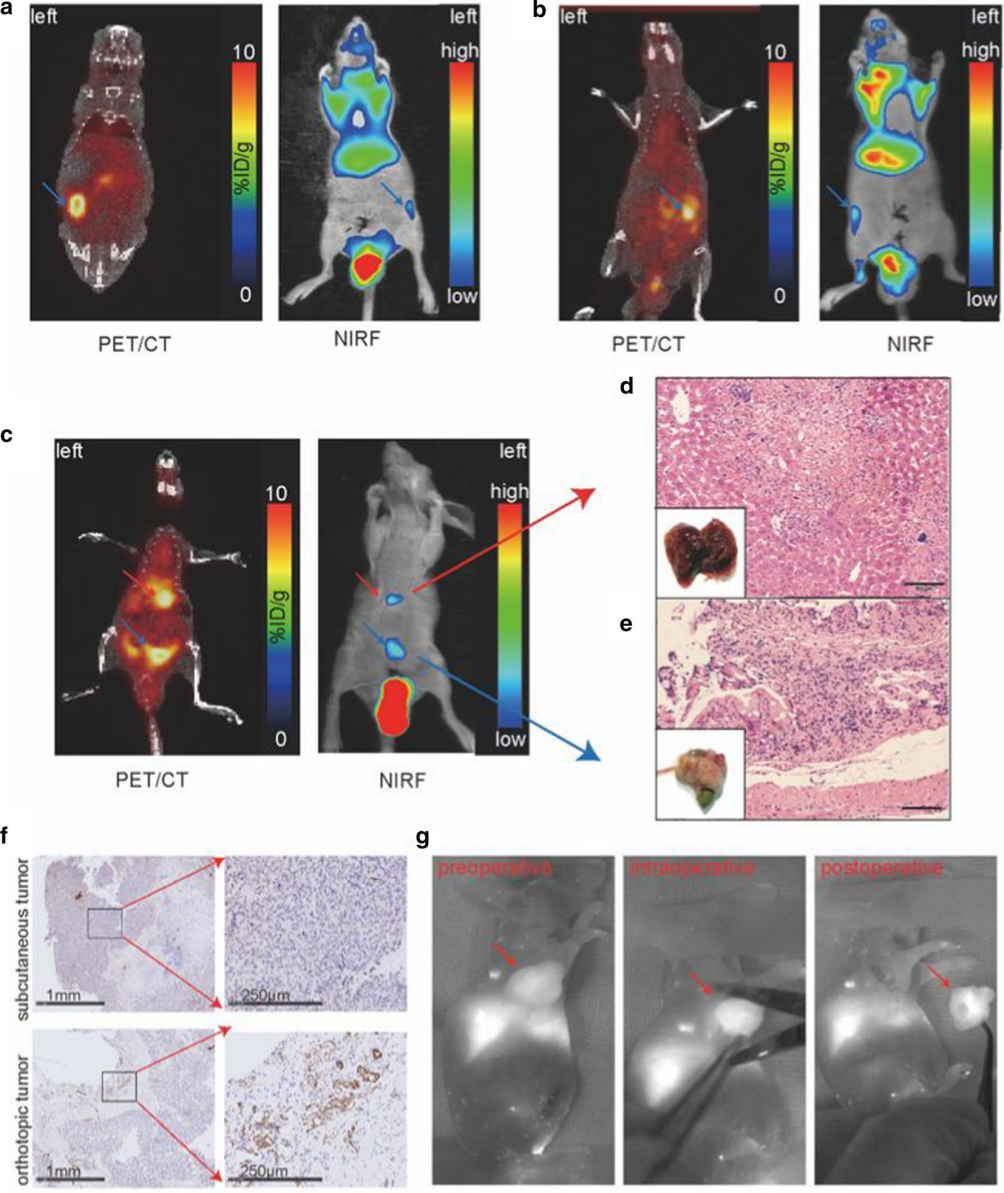
Multimodal PET/CT/NIRF images of orthotopic colon cancer and pre-, intra- and postoperative NIRF images. Multimodal PET/CT and NIRF images of the left (**a**) and right (**b**) orthotopic colon cancer model. The blue arrows denote colon tumors in situ. **c** Multimodal PET/CT and NIRF images of orthotopic colon cancer model (blue arrows) with liver metastasis (red arrows). **d** The visual observation of tumors and HE staining of pathological sections (scale bar: 50 μm). **f** Immunohistochemistry assay of CD31 in orthotopic and subcutaneous colon cancer. **g** Representative NIRF images of tumor-bearing mice pre-, intra-, and postoperatively. The red arrows point to the tumor

For further studying the nanoprobe, orthotopic colon cancer was used as models for multi-modal imaging. Figure 6 shows that the tumor is clearly visible not only in the primary lesion (right abdomen, Fig. 6a, b), but also in a metastatic site (liver, Fig. 6c). Histology with HE staining confirmed the colon cancer and liver metastasis (Fig. 6d, e). Orthotopic colon cancer showed higher expression level of CD31, while subcutaneous colon tumor tissues showed lower expression of CD31 (Fig. 6f).

### Real-time NIRF imaging for intraoperative guidance

Having done Multi-modal animal PET/CT and NIRF imaging of orthotopic colon cancer, we further performed the tumor surgery under real-time NIRF imaging. Under the guidance of real-time NIRF imaging, the location of the tumor could be identified preoperatively, the boundary of the tumor can be confirmed intraoperatively, and the absence of residual tumor can be observed postoperatively (Fig. 6g). The image-guided video is shown in Additional file 1: Video S1.

### In vivo toxicity studies

**Fig. 7:**
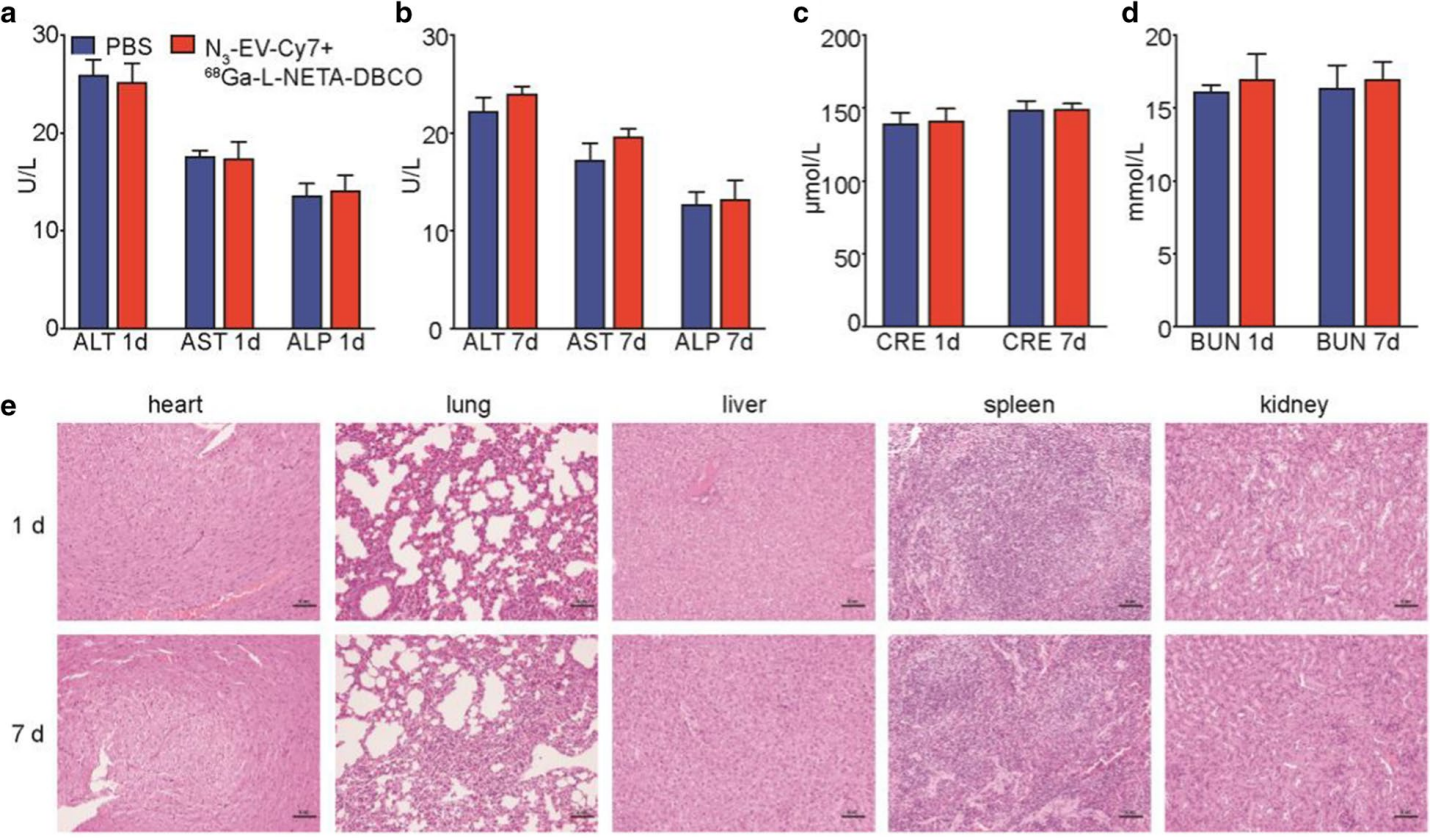
In vivo toxicity evaluation by blood test and histology analysis. **a**–**d** Liver function makers (ALT, AST and ALP) and kidney function markers (BUN and CRE) after i.v. injection with N_3_-EV-Cy7 + ^68^ Ga-L-NETA-DBCO over 1 d and 7 d. **e** Representative H&E staining images of major organs from the euthanized mice. Bar = 50 μm. All bars represent as means ± SD (n = 4)

BALB/c mice (n = 5) received an i.v. injection of N_3_-EV-Cy7 + ^68^ Ga-L-NETA-DBCO or PBS to evaluate the in vivo potential toxicity. No significant hepatic or renal toxicity was observed from the indicating normal values of liver and kidney function markers, including ALT, AST, ALP, BUN and CRE (Fig. 7a–d). We did not observe significantly evidence of major organ damage from the H&E staining sections (Fig. 7e).

## Discussion

In this study, we prepared a multi-modal nanoprobe with extracellular vesicles as the nanocarrier. This nanoprobe exhibited suitable size, good stability, and was non-cytotoxic and bound well to tumors. Multi-modal imaging with PET and NIRF were successfully achieved with this nanoprobe, and the surgery was conducted successfully under real-time NIRF imaging. To the best of our knowledge, pre-targeting strategy have not been applied for labeling extracellular vesicles-based nanoprobe. In other words, this is the first report of extracellular vesicles as the nanocarriers for multi-modal imaging and surgery of tumors with the application of pre-targeting strategy.

Multimodality imaging, a combination of two or more imaging modalities, can provide more complementary information for imaging than individual techniques. To date, many researchers have constructed multimodality nanoprobes with the application of synthetic nanoparticles, such as liposomes, metal nanoparticles, and magnetic nanoparticles, because of their advantages, such as large carrying capacity and facile surface modification [23, 24]. However, the widespread use of artificial drug carriers has been prevented by their potential toxicity, immunogenicity, and inability to penetrate and target specific organs. These disadvantages may be largely avoided when these drug carriers are in the form of biological structures.

The use of extracellular vesicles as nanocarriers has attracted wide attention because of their excellent biocompatibility [25]. Extracellular vesicles could facilitate their extravasation through tumor vessels and their subsequent diffusion into tumor tissues. They have the advantages of membrane-permeability, good stability in vivo, easy surface modification, and large loading capacity. Most importantly, unlike extracellular vesicles from cancer cells, normal cell-derived extracellular vesicles almost have no toxicity and are well tolerated by human [26]. Therefore, extracellular vesicles are favorable candidate nanocarriers. In this study, our data also confirmed the advantages of the extracellular vesicles-based nanoprobe. The extracellular vesicles-based nanoprobe was stable as revealed by its unchanged size and zeta potential over 8 d. The nanoprobe exhibited no obvious cytotoxicity, as shown by CCK8 assay and in vivo toxicity studies. Additionally, fluorescence imaging and confocal imaging showed that extracellular vesicles had great tumor cell binding ability and excellent cell internalization. Therefore, extracellular vesicles-based nanoprobes have great promise for multi-modal imaging.

As we all know, the half-life of ^68^ Ga is short (67.7 min), which does not match the ADSCs-EV with high molecular weight. According to the report [27], pre-targeting strategy can combine short half-life nuclide and long half-life molecular. Therefore, the pre-targeting strategy based on click reaction (N_3_-DBCO) was applied in this study [28]. The results showed a maximum tumor uptake when the pre-targeting time was 20 h and the imaging time was 2 h. Low uptakes in liver and spleen were displayed at all time points compared with the study by Shi et al. [22]. Although PET/CT imaging offered an abundance of information needed for diagnosis and preoperative planning, it cannot be applied for intraoperative detection of tumor tissues.

Optical image-guided surgery provides the accurate analysis of biological lesions through optical molecular probes, allowing more precise surgery. Compared with traditional optical imaging, NIRF imaging has been applied for intraoperative tumor resection studies because of some advantages, such as high temporal resolution, super sensitivity and lower tissue auto-fluorescence. After comparing the NIRF images acquired at different time periods, the appropriate imaging time for image-guided surgery has been suggested to be 10–30 h. Our surgery was performed under real-time NIRF imaging to further confirm the functions of this nanoprobe. During the operation, we could observe the tumor location, tumor boundaries, and residual after tumor resection clearly.

The orthotopic colon cancer model was designed to demonstrate the advantages of the multimodal nanoprobe further. The biological characteristics of orthotopic colon cancer are more similar to human tumors than subcutaneous colon cancer, such as the position, the morphology, the surrounding tissues, the blood supply, and the environment of colorectal tumors [29]. Excitingly, the orthotopic transplantation tumors showed higher tracer uptake than the subcutaneous tumors in our study according to the PET images. According to CD31 expression, the orthotopic tumor tissues exhibited higher density of blood vessels than subcutaneous tumor tissues.

Some issues remain to be addressed in this study. First, the production and isolation of extracellular vesicles remain challenging. For example, the differential centrifugation method we applied in this study has the advantages of high yield and low cost, but this method cannot be applied in the clinic because of the low purity and complex procedures. The extracellular vesicles from ADSCs are also limited by low yield and cannot be employed in clinical applications. Recently, some researchers tried to isolate extracellular vesicles from milk and fruits to increase the yield [30]. Second, the composition, function, specific targeting, and metabolism in vivo of extracellular vesicles have not yet been fully elucidated. Additionally, various DSPE-PEG_2000_ functionalized head-groups can be modified on extracellular vesicles to increase the tumor-targeting ability of the nanoprobe using hydrophobic interaction approach. Briefly, further in vitro and in vivo animal studies based on extracellular vesicles as a nanocarrier are required before clinical translation.

## Conclusion

In this study, a novel extracellular vesicles-based nanoprobe was successfully engineered for multimodal PET/CT/NIRF imaging and image-guided surgery of colon cancer. This nanoprobe showed promise for preoperative evaluation and intraoperative surgical guidance. Our data verified that a pre-targeting strategy can be applied to gain high quality images. This research also proved that extracellular vesicles are also potential high-quality nanocarriers for multimodality imaging and have broad application prospects.

## Materials and methods

### Cell culture

This study was approved by the Ethics Committee at the Tongji Medical College of Huazhong University of Science and Technology (No. 2018-S288). ADSCs were isolated from the subcutaneous fat from patients without cancers. The adipose tissue was cut into small pieces and the connected fascial tissue was removed. Then the adipose tissue was digested for 1 h with 0.2% collagenase type I (Sigma, St Louis MO, USA) and centrifuged for 4 min at 1000 rpm. The cell pellets obtained were passed through a 70-μm filter (Corning, Rochester NY, USA), and then cultivated in Dulbecco’s modified Eagle’s medium (DMEM; Gibco, Gaithersburg MD, USA) with 10% fetal bovine serum (FBS, Serapro, Naila, Germany). The human colon cancer cell line HCT116 and mouse colon cancer CT26 were preserved in our laboratory (Hubei Province Key Laboratory of Molecular Imaging) and propagated in an RPMI-1640 medium (Gibco) supplemented with 10% FBS.

### ADSCs-EV isolation

Extracellular vesicles from serum-free ADSCs culture supernatant were obtained by differential centrifugation. Dead cells and cell fragments were removed by centrifugation at 3000 g for 30 min. Then the supernatants were centrifuged at 13,000 g for 70 min. The supernatants were concentrated by an Amicon® Ultra-15 Centrifugal Filter Device (100 kDa molecular weight, Millipore, USA). Finally, the supernatants were centrifuged at 120,000 g for 70 min to obtain ADSCs-EV, which were subsequently suspended in PBS, passed through a 0.22 μm filter, and quantified by surface proteins with a BCA Protein Assay Kit (Beyotime, Shanghai, China) and stored at − 80 °C.

### ADSCs-EV characterization

Extracellular vesicles were examined by transmission electron microscopy (TEM, Hitachi, Japan) and dynamic light scattering (DLS, Malvern Instruments Ltd., Worcestershire, UK). Then western blot was carried out to identify the ADSCs-EV by several surface markers. Briefly, equal amounts of total protein samples from ADSCs-EV (30 µg) were loaded in each well of SDS-PAGE gels. The gels were subsequently transferred onto PVDF membranes and incubated overnight at 4 °C with following primary antibodys: CD9 (Cat #A1703; ABclonal), GM130 (Cat #11308‐1‐AP; Proteintech), CD63 (Cat #ab134045; Abcam) and β‐tublin (Cat #10094‐1‐AP; Proteintech). On the following day, the membranes were incubated with HRP-conjugated antibody (Aspen, China) for 1 h. After incubation, the membranes were again washed 3 times with PBS and exposed to X-ray films using ECL detection reagents (#WP20005, Thermo Fisher). Changes in hydrodynamic diameters were monitored for 8 d by DLS to test the stability of the ADSCs-EV in vitro. A Cell Counting Kit-8 (CCK8) kit (SAB, College Park, MD, USA) was used to identify the cytotoxicity of different concentrations of ADSCs-EV and ^68^ Ga-L-NETA-DBCO in HCT116 human colon cancer cells.

### In vitro cell binding

The modification of extracellular vesicles was adapted from a literature reported method [31]. Briefly, we introduced 1 mg DSPE-PEG per 1 mg extracellular vesicles. DSPE-PEG_2000_-Cy5, DSPE-PEG_2000_-N_3_ (Ruixi, Xi’an, China) and ADSCs-EV were incubated at room temperature for 30 min, and Cy5-EV-N_3_ were obtained. Cy5-EV-N_3_ (100 μg /mL) were added onto HCT116 cells grown in a confocal dish and incubated at 37℃ for different time periods (10, 20, and 30 h). The cell nuclei were counterstained with 4′,6-Diamidino-2-phenylindole (DAPI) (Boster, Wuhan, China). Cells were fixed with paraformaldehyde and observed under a Fluorescence microscope. Confocal microscopy was also used to observe the tumor-binding ability. Cy5-EV-N_3_ (100 μg/mL) were added into the HCT116 cells cultured in a confocal dish and incubated for 30 h. Additionally, the skeleton of tumor cells was stained with FITC-phalloidin, and nuclei were stained with 4′,6-Diamidino-2-phenylindole (DAPI; Boster, Wuhan, China). Finally, the confocal dish was fixed with paraformaldehyde and observed by confocal microscopy (LSM 880, ZEISS).

### Tumor-bearing nude mouse models

The protocol of mouse experiments was reviewed and approved by the Animal Care Committee of Tongji Medical College, Huazhong University of Science and Technology. HCT116 cells (5 × 10^6^) suspended in 100 μL PBS were subcutaneously injected into the right upper limb of BALB/C nude mice (male, 4 weeks old, Beijing HFK Bioscience co., Ltd, China). After the tumor size reached approximately 0.8 cm, the mice were prepared for study. Orthotopic colon cancer models were also prepared with the following protocol. The 5-week BALB/C nude mice were laparotomized to expose the cecum, then HCT116 cells (5 × 10^6^) suspended in 50 μL PBS were injected into the serosal layer of the colon. Four weeks after cell injections, the mice were prepared for study.

### The modification of ADSCs-EV

Using hydrophobic insertion approach [31], DSPE-PEG_2000_-Cy7 (Ruixi, Xi’an, China) and DSPE-PEG_2000_-N_3_ (Ruixi, Xi’an, China) were incubated with ADSCs-EV for 30 min at 37 °C to form N_3_(Cy7)-PEG_2000_-DSPE-ADSCs-EV (Cy7-EV-N_3_). The samples were passed through centrifugal filter devices (100 kDa molecular weight, Amicon®Ultra-15) before further use.

### Synthesis and identification of ^68^ Ga-L-NETA-DBCO

^68^GaCl_3_ was obtained from the ^68^Ge/^68^ Ga generator with HCl (0.05 M) as eluent. Sodium acetate (1.25 M, pH = 8.6) was added to 500 μL of ^68^GaCl_3_ (187 MBq) to adjust the pH to 3.7. L-NETA-DBCO (5 nmol) was used to chelate the radionuclide ^68^ Ga, and the reaction was maintained 10 min at 100 °C. After the mixture was cooled, a C18 column was used to purify ^68^ Ga-L-NETA-DBCO. ^68^ Ga-L-NETA-DBCO was conjugated with N_3_-modifed ADSCs-EV by in vivo click reaction, which enables PET imaging. The radiochemical purity and in vitro stability of the probe (2 h in fetal bovine serum) were measured by high-performance liquid chromatography (HPLC).

### Cellular uptake

To assess cell uptake, we incubated 1 × 10^6^ of HCT116 cells with RPMI-1640 medium supplemented with 10% fetal bovine serum containing Cy7-EV-N_3_ (100 μg/mL) at 37 °C for 0, 10, 20, and 30 h. Then the supernatants were removed. ^68^ Ga-L-NETA-DBCO (37 kBq/well) were added to the HCT116 cells. After incubation for 1, 2, and 3 h, the supernatants were removed and washed with PBS. The remaining cells were lysed in NaOH. Cell lysates and supernatants were collected. Radioactivity was measured using a γ-counter (PerkinElmer, USA).

### In vivo PET imaging

In order to identify the best time points, Cy7-EV-N_3_ (200 μg) was intravenously injected into the HCT116 tumor-bearing nude mice at different pre-targeting time points (10, 20, and 30 h). ^68^ Ga-L-NETA-DBCO (3.7 MBq) were injected into the mice (n = 3 per group) via the tail vein. Mice were anesthetized with 2% isoflurane, and micro-PET static imaging was performed at 1, 2 and 3 h after the injection of ^68^ Ga -L-NETA-DBCO. Static PET images were collected for 10 min using a small-animal PET scanner (TransPET BioCaliburn 700, Raycan Technology Co., Ltd, Suzhou, China). PET images were reconstructed with the ordered-subset expectation maximization three-dimensional/maximum a posteriori probability algorithm, and then image analysis was performed using Amide (http://amide.sourceforge.net) and Carimas 2.10 (Turku PET Centre, Finland) software.

### NIRF imaging

Cy7-EV-N_3_ was injected into the mice bearing HCT116 tumor grafts (n = 3 per group) via the tail vein for NIRF imaging. Mice were anesthetized with 2% isoflurane, and NIRF imaging was performed at different times (1, 5, 10, 20, 30, and 50 h). The static NIRF images were acquired with 750-nm excitation and 790-nm emission filters using an IVIS spectrum imaging system (In-Vivo FX PRO, Bruker, Germany). NIRF images were analyzed by Bruker MI (Germany).

### Multimodal PET/CT and NIRF imaging of orthotopic colon transplantation tumor

Twenty hours after Cy7-EV-N_3_ injection, ^68^ Ga-L-NETA-DBCO were injected into the orthotopic colon transplantation tumor model mice via the tail vein for click chemistry in vivo. 2 h after ^68^ Ga-L-NETA-DBCO injection, the mice’s bladders were emptied by compression and the mice were anesthetized using 2% isoflurane. Static PET/CT images were collected for 10 min using a small-animal PET scanner (TransPET Discoverist 180, Raycan Technology Co., Ltd, Suzhou, China). PET/CT images were reconstructed with the ordered-subset expectation maximization three-dimensional/maximum a posteriori probability algorithm, and then the analysis of images was done using Amide (http://amide.sourceforge.net) and Carimas 2.10 software. After PET/CT imaging was completed, NIRF imaging was subsequently performed and analyzed as described above. The mice were then sacrificed and the tumor tissue was collected for HE staining of pathological sections.

### Immunohistochemistry analysis

The CD31 immunohistochemistry analysis was performed to evaluate the vascularization of the tumors. The HCT116 tumor tissues were collected, fixed in 4% paraformaldehyde and embedded in paraffin. The tumor Sects. (5 μm) were dewaxed, rinsed with EDTA buffer (pH 9.0), and blocked with 3% hydrogen peroxide. The tumor sections were incubated with anti-CD31 antibody (Abcam, Cambridge MA, USA) at 4 °C overnight. Then the tumor slices were incubated with secondary antibody (HRP-labeled goat anti-rabbit IgG, Abbkine, Redlands CA, USA) at room temperature for 30 min. The sections were stained with 3, 3’-diaminobenzidine (DAB, Beyotime, Hangzhou, China) for 8 min, subsequently by counterstaining with hematoxylin (Beyotime) for 2 min and were observed under microscopy.

### Real-time NIRF imaging for intraoperative guidance

Twenty hours after the injection of Cy7-EV-N_3_, resection of the subcutaneous tumors in mice was performed using a real-time IVIS spectrum imaging system (Premium Imaging FB800, Premium imaging, California, USA).

### In vivo* toxicity studies*

BALB/c mice (n = 4 per group) received an i.v. injection of N_3_-EV-Cy7 (200 μg) + ^68^ Ga-L-NETA-DBCO (7.4 MBq) or PBS. Mice were euthanized on 1^st^ and 7^th^ day after the injection. Their blood samples and major organs were collected. The function of liver and kidney, such as alanine amino transferase (ALT), aspartate aminotransferase (AST), and alkaline phosphatase (ALP), blood urea nitrogen (BUN), and creatinine (CRE) were measured by the blood biochemical autoanalyzer (Chemray 240, Rayto Life and Analytical Sciences Co., Ltd, China). Hematoxylin and eosin (H&E) of major organs (hearts, livers, spleens, lungs and kidneys) were examined using an optical microscope (IX73, Olympus, Japan).

### Statistical analysis

Data are shown as the mean ± standard deviation (SD). Comparisons between groups were evaluated with the unpaired Student’s *t*-test. *p* < 0.05 was considered to be statistically significant. Statistical analysis was conducted using GraphPad Prism v8.0 software.

## Supplementary Information

**Additional file 1: Video S1.** The real-time NIRF imaging guided tumor surgery.

**Additional file 2**: **Figure S1**. Radiolabeling efficiency and stability. A The radiolabeling efficiencies of 68Ga-L-NETA-DBCO. B The stability of 68Ga-L-NETA-DBCO. **Figure S2**. PET imaging of the control group. A Representative static PET images at 1 h after the injection of 68Ga-L-NETA-DBCO. B Representative static PET images at 2 h after the injection of 68Ga-L-NETA-DBCO. White arrows point the tumor sites. **Table S1** The HCT116 cell and ADSCs after 24 h incubation with ADSCs-EV at different concentrations (μg/mL). Bars represent means ± SD (n = 4). **Table S2**. The HCT116 cell and adipose stem cell viability after incubation with ADSCs-EV at different time points (h). Bars represent means ± SD (n = 4). **Table S3**. The HCT116 cell and adipose stem cell viability after incubation with 68Ga-L-NETA-DBCO at different time points (h). Bars represent means ± SD (n = 4).

## Data Availability

Not applicable.

## Abbreviations

EV: Extracellular vesicles
ADSCs: Adipose-derived stem cells
PET/CT: Positron emission tomography/computed tomography
MR: Magnetic resonance
NIRF: Near-infrared fluorescence
ADSCs-EV: Extracellular vesicles extracted from adipose-derived stem cells
Cy7: Cyanine7
DSPE-PEG_2000_-Cy7: Cy7-terminated 1,2-distearoyl-sn-glycero-3-phosphetha-mine-[(polyethyleneglycol)-2000]
TEM: Transmission electron microscopy
DLS: Dynamic light scattering
CCK8: Counting Kit-8
Cy5: Cyanine5
DSPE-PEG_2000_-Cy5: Cy5 labeled DSPE-PEG_2000_
DSPE-PEG_2000_-N_3_: N_3_ labeled DSPE-PEG_2000_
EV-Cy5: ADSCs-EV labeled with Cy5
DAPI: 4′,6-Diamidino-2-phenylindole
SPECT: Single-photon emission computed tomography
FBS: Fetal bovine serum
PBS: Phosphate buffer saline
SD: Mean ± standard deviation
T/M ratios: Tumor-to-muscle ratios
T/L ratios: Tumor-to-liver ratios
%ID/g: Injected dose per gram
ALT: Alanine amino transferase
AST: Aspartate aminotransferase
ALP: Alkaline phosphatase
BUN: Blood urea nitrogen
CRE: Creatinine
H&E: Hematoxylin and eosin
