# Supplementary material for: Extracellular vesicles-based pre-targeting strategy enables multi-modal imaging of orthotopic colon cancer and image-guided surgery
Source: J Nanobiotechnology. 2021 May 22;19:151. doi: 10.1186/s12951-021-00888-3 (PMC8141172; doi:10.1186/s12951-021-00888-3)
Supplement: Supplementary file 2 — Additional file 2: Figure S1. Radiolabeling efficiency and stability. A The radiolabeling efficiencies of 68Ga-L-NETA-DBCO. B The stability of 68Ga-L-NETA-DBCO. Figure S2. PET imaging of the control group. A Representative static PET images at 1 h after the injection of 68Ga-L-NETA-DBCO. B Representative static PET images at 2 h after the injection of 68Ga-L-NETA-DBCO. White arrows point the tumor sites. Table S1 The HCT116 cell and ADSCs after 24 h incubation with ADSCs-EV at different concentrations (μg/mL). Bars represent means ± SD (n = 4). Table S2. The HCT116 cell and adipose stem cell viability after incubation with ADSCs-EV at different time points (h). Bars represent means ± SD (n = 4). Table S3. The HCT116 cell and adipose stem cell viability after incubation with 68Ga-L-NETA-DBCO at different time points (h). Bars represent means ± SD (n = 4). [file 12951_2021_888_MOESM2_ESM.docx]

Title page: Extracellular vesicles-based pre-targeting strategy enables multi-modal imaging of orthotopic colon cancer and image-guided surgery

Boping Jing^a#^, Ruijie Qian^a#^, Dawei Jiang^a^, Yongkang Gai^a^, Zhen Liu^a^, Feng Guo^b^, Sen Ren^c^, Yu Gao^a^, Xiaoli Lan ^a,^*, Rui An^a,^*.

^a^ Department of Nuclear Medicine, Union Hospital, Tongji Medical College, Huazhong University of Science and Technology, Wuhan 430022, China

^b^ Department of Pancreatic Surgery, Union Hospital, Tongji Medical College, Huazhong University of Science and Technology, Wuhan 430022, China

^c^ Department of Hand Surgery, Union Hospital, Tongji Medical College, Huazhong University of Science and Technology, Wuhan 430022, China

#Boping Jing and Ruijie Qian contributed equally to the manuscript.

***Corresponding Authors:** Rui An, No. 1277 Jiefang Ave, Wuhan, Hubei Province 430022, China. Phone: +86-13986113240; Fax: +86-27-87543437. E-mail: [1975xh0577@hust.edu.cn](mailto:1975xh0577@hust.edu.cn)

Xiaoli Lan, No. 1277 Jiefang Ave, Wuhan, Hubei Province 430022, China. Phone: +86-13886193262; Fax: +86-27-85726282. E-mail: [LXL730724@hotmail.com](mailto:LXL730724@hotmail.com);

Figure S1. Radiolabeling efficiency and stability. A The radiolabeling efficiencies of ^68^Ga-L-NETA-DBCO. B The stability of ^68^Ga-L-NETA-DBCO.


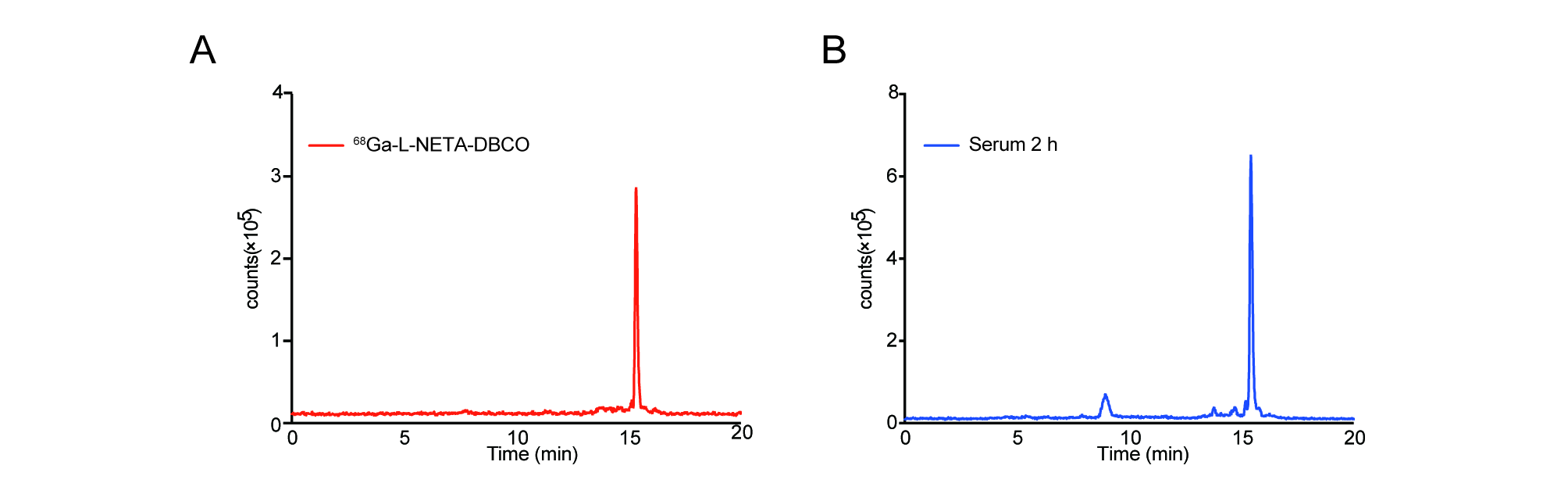


Figure S2. PET imaging of the control group. A Representative static PET images at 1 h after the injection of ^68^Ga-L-NETA-DBCO. B Representative static PET images at 2 h after the injection of ^68^Ga-L-NETA-DBCO. White arrows point the tumor sites.


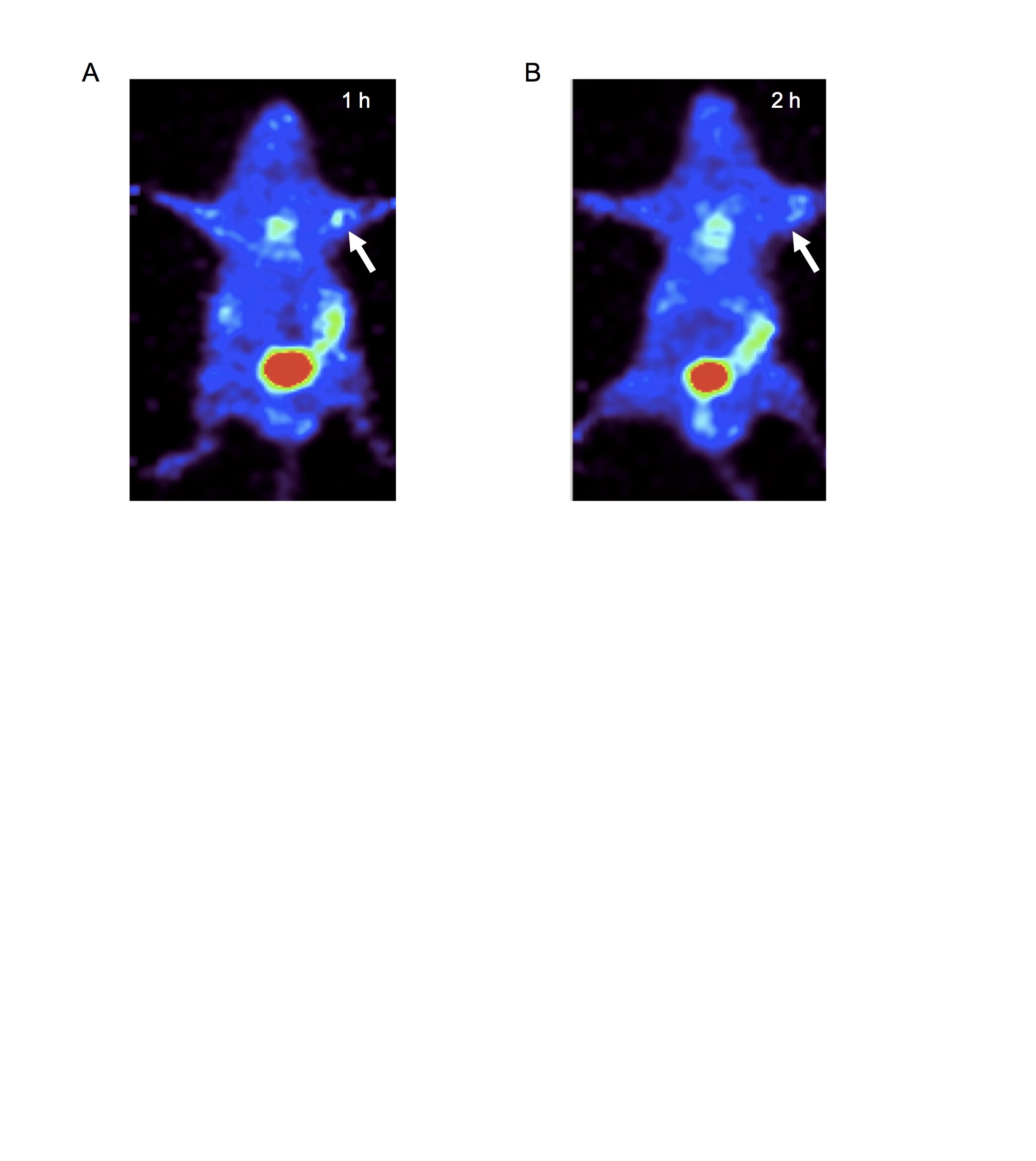


Table S1 The HCT116 cell and ADSCs after 24 h incubation with ADSCs-EV at different concentrations (μg/mL). Bars represent means ± SD (n = 4).

|  | 3.125 | 6.25 | 12.5 | 25 | 50 | 100 |
| --- | --- | --- | --- | --- | --- | --- |
| HCT116 | 0.98±0.04 | 0.97±0.04 | 1.01±0.01 | 1.02±0.05 | 0.98±0.06 | 0.99±0.02 |
| ADSCs | 0.97±005 | 0.98±0.09 | 0.99±0.10 | 0.97±0.07 | 0.98±0.06 | 0.97±0.05 |

Table S2. The HCT116 cell and adipose stem cell viability after incubation with ADSCs-EV at different time points (h). Bars represent means ± SD (n = 4).

|  | 0 | | 2 | 6 | 12 | 18 | 24 | 48 | 72 |
| --- | --- | --- | --- | --- | --- | --- | --- | --- | --- |
| HCT116 | | 1.01±0.07 | 0.97±0.03 | 0.94±0.02 | 0.96±0.02 | 0.94±0.04 | 0.99±0.01 | 0.96±0.03 | 0.97±0.04 |
| ADSCs | 1.00±0.07 | | 0.97±0.11 | 1.00±0.06 | 0.99±0.05 | 0.94±0.04 | 0.99±0.03 | 1.02±0.02 | 1.04±0.05 |

Table S3. The HCT116 cell and adipose stem cell viability after incubation with 68Ga-L-NETA-DBCO at different time points (h). Bars represent means ± SD (n = 4).

|  | 0 | | 2 | 6 | 12 | 18 | 24 | 48 | 72 |
| --- | --- | --- | --- | --- | --- | --- | --- | --- | --- |
| HCT116 | | 0.99±0.03 | 0.96±0.1 | 0.94±0.07 | 0.98±0.08 | 0.97±0.4 | 0.98±0.2 | 0.98±0.06 | 0.99±0.15 |
| ADSCs | 0.98±0.06 | | 0.98±0.13 | 1.01±0.03 | 0.93±0.06 | 0.95±0.05 | 1.01±0.07 | 0.96±0.02 | 0.94±0.08 |
